# Focal adhesion kinase inhibition enhances response to checkpoint immunotherapy in hepatocellular carcinoma

**DOI:** 10.1515/jtim-2025-0051

**Published:** 2025-12-12

**Authors:** Xinyu Liu, Fangyanni Wang, Peng Cui, Ziyi Zheng, Ning Zhang, Ruirui Kong

**Affiliations:** Translational Cancer Research Center, Peking University First Hospital, Beijing, China; International Cancer Institute, School of Basic Medical Sciences, Peking University Health Science Center, Beijing, China; Chinese Institutes for Medical Reaearch, Capital Medical University, Beijing, China; Yunnan Baiyao Group, Kunming, Yunnan Province, China

**Keywords:** hepatocellular carcinoma, focal adhesion kinase, immune checkpoints inhibitors, CD8^+^ T cells, PD-1

## Abstract

**Background and objectives:**

Despite the remarkable efficacy of immune checkpoint inhibitors (ICIs) in cancer therapy, their clinical benefit in hepatocellular carcinoma (HCC) remains limited. Focal adhesion kinase (FAK) plays a pivotal oncogenic role in various tumors by promoting angiogenesis, tumor proliferation, and immunosuppression. Therefore, targeting FAK represents a promising strategy to enhance immunotherapy outcomes in HCC.

**Methods:**

We analyzed RNA-sequencing data from The Cancer Genome Atlas (TCGA) and Gene Expression Omnibus (GEO) to compare *PTK2* (encoding FAK) expression between HCC tumors and adjacent normal tissues. Associations between *PTK2* expression levels and clinicopathological features were systematically evaluated. Immune cell infiltration landscapes were characterized using CIBERSORT and ssGSEA algorithms, while the Tumor Immune Dysfunction and Exclusion (TIDE) computational framework was applied to predict HCC responsiveness to ICIs based on *PTK2* expression. To experimentally validate therapeutic efficacy, we established orthotopic liver cancer models by transposon-mediated integration of Myc and Kras^G12D^ oncogenes into hepatocytes of Trp53 null/null mice (pTMK/Trp53^-/-^), coupled with subcutaneous xenograft models. These models were treated with FAK inhibitor IN10018 as monotherapy or in combination with anti-PD-1 immunotherapy.

**Results:**

FAK was highly expressed and frequently amplified in HCC tumors, which predicted worse pathological features of patients. A notable feature of FAK-positive HCC tumors was an adverse immune microenvironment marked by a depletion of CD8^+^ cytotoxic T cells and an abundance of suppressive myeloid cells. Pharmacologic FAK inhibition demonstrated efficacy against primary liver cancer (PLC) tumors in both orthotopic and subcutaneous mouse models and was associated with progressive reduction in fibrosis and angiogenesis and stimulation of cytotoxic CD8^+^ T cell function. Synergy with anti-PD-1 blockade substantially reprogrammed the immune microenvironment, leading to tumor regression, compared to current therapeutic strategies for HCC.

**Conclusions:**

FAK inhibitors can enhance the sensitivity of HCC to anti-PD-1 therapy by inhibiting angiogenesis and fibrosis and promoting CD8^+^ T cell infiltration. This effect exceeds the efficacy of the current first-line treatment, highlighting FAK inhibition as a novel and promising therapeutic strategy for HCC.

## Introduction

Primary liver cancer (PLC) remains a critical global health burden, ranking as the sixth most common malignancy and the third leading cause of cancer-related mortality worldwide.^[1,2]^ Hepatocellular carcinoma (HCC) accounts for 80% and is often treated by surgical resection, orthotopic liver transplantation, or local percutaneous tumor ablation. Unfortunately, most patients with HCC diagnosed at an advanced stage have limited therapy options.^[3,4]^ Systemic pharmacotherapy serves as the cornerstone for managing advanced HCC, such as Sorafenib and Lenvatinib, which have been approved as first-line therapies. Yet, these multi-kinase inhibitors exhibit limited durability of response and confer suboptimal survival benefits for patients.^[5, 6, 7]^ This therapeutic stagnation underscores the urgent need for innovative strategies to redefine clinical outcomes for patients with HCC.

The advent of immune checkpoint inhibitors (ICIs) has revolutionized immune therapy for advanced HCC,^[8]^ with landmark trials demonstrating clinical benefits from both monotherapy and combination regimens.^[9, 10, 11, 12]^ Notably, the United States Food and Drug Administration (FDA)-approved tyrosine kinase inhibitor (TKI)-based ICI (atezolizumab/bevacizumab) combination was granted accelerated approval by FDA for HCC treatment in first-line therapy based on IMbrave150 trial results.^[12]^ However, therapeutic limitations persist as approximately 70% of patients exhibit primary resistance or acquired unresponsiveness to current ICI-based therapies.^[12]^ This stark reality necessitates a dual strategy: (1) identification of robust predictive biomarkers for patient selection, and (2) development of rationally designed combination therapies to ultimately improve patients’ survival.^[13,14]^ Such approaches could unlock the full potential of immunomodulation in HCC management.

Focal adhesion kinase (FAK), encoded by the *PTK2* gene, is a non-receptor tyrosine kinase that orchestrated adhesion-mediated signaling to regulate critical oncogenic processes including cell survival, migration, and metastatic invasion.^[15]^ FAK is frequently highly expressed across multiple malignancies and associated with angiogenesis, therapy resistance, and immunosuppression within the tumor microenvironment.^[16,17]^ Recent studies demonstrated that pharmacologic inhibition of FAK has exhibited a promising antitumor activity in some cancers, but HCC remains an outlier in this therapeutic revolution and the mechanistic basis of which requires further study.

In this study, we developed a genetically engineered orthotopic liver cancer model through Myc overexpression, KRAS^G12D^ activation in Trp53 liver conditional knockout mice, which recapitulated human PLC.^[18]^ Using a GEMM coupled with subcutaneous xenografts derived from HCC cell lines, we systematically conducted assessments of IN10018 (a high-selectivity FAK inhibitor),^[19]^ including its antitumor efficacy and mechanistic underpinnings. FAK inhibition potentiates responsiveness to immunotherapy with PD-1 blockade. Then, synergistic therapy was further benchmarked against the first-line standard (VEGFR-TKI/ PD-1 inhibitor combinations), providing robust preclinical evidence to refine immune-combination strategies for PLC patients.

## Materials and methods

### Data download and processing

For The Cancer Genome Atlas-Liver Hepatocellular Carcinoma (TCGA-LIHC) cohort, Ribonucleic Acid (RNA) sequencing data (normalized as transcripts per million, TPM) and corresponding clinical records were obtained from TCGA portal (https://portal.gdc.cancer.gov/) and the UCSC Xena platform (https://xenabrowser.net/), respectively.^[20]^ Patients with survival durations shorter than 30 days were excluded, yielding a final cohort of 340 HCC patients and 50 normal liver tissue samples. Validation cohorts from the GEO (https://www.ncbi.nlm.nih.gov/geo/) included the GSE14520 dataset (242 tumor samples, comprising 233 HCC specimens paired with adjacent normal tissues) and the GSE235863 dataset (bulk RNA-seq data from 15 HCC patients stratified by treatment response: 11 responders (6 partial responders [PR], 5 complete responders [CR]) and 4 non-responders). The detailed clinical pathological characteristics of the liver cancer cohort, including gender, age, tumor stage, and *PTK2* expression levels, have now been incorporated into Table 1.

**Table 1:** Univariate and multivariate Cox analysis of *PTK2* in TCGA cohort

Characteristics	Total (*N*)	HR (95% CI) Univariate analysis	*P* value	HR (95% CI) Multivariate analysis	*P* value
Age	340				
>65	124	Reference			
≤65	216	0.824 (0.576-1.180)	0.292		
Gender	340				
Female	108	Reference			
Male	232	0.819 (0.569-1.179)	0.283		
Stage	319				
Stage I/II	238	Reference		Reference	
Stage III/IV	81	2.762 (1.887-4.043)	<0.001	2.083 (0.284-15.257)	0.470
T. stage	337				
T1/2	252	Reference		Reference	
T3/4	85	2.848 (1.985-4.085)	<0.001	1.515 (0.206-11.143)	0.683
N. stage	240				
N0	237	Reference			
N1	3	2.147 (0.525-8.776)	0.288		
M. stage	246				
M0	243	Reference		Reference	
M1	3	4.288 (1.345-13.668)	0.014	3.106 (0.927-10.405)	0.066
*PTK2*	340	1.030 (1.012-1.049)	<0.001	1.042 (1.021-1.063)	<0.001

TCGA: The Cancer Genome Atlas.

### Tumor immune microenvironment evaluation

The tumor microenvironment was interrogated using the ESTIMATE algorithm to compute stromal, immune, and combined estimate scores.^[21]^ Immune landscape profiling of the TCGA cohort was performed through two complementary approaches: (1) single-sample gene set enrichment analysis (ssGSEA) implemented *via* the “GSVA” R package quantified infiltration levels of 28 distinct immune cell populations,^[22]^ and (2) the CIBERSORT algorithm (executed using the “CIBERSORT” R package) deconvoluted relative proportions of 22 immune cell subsets.^[23]^ To assess potential immune evasion mechanisms, the Tumor Immune Dysfunction and Exclusion (TIDE) platform (http://tide.dfci.harvard.edu/) was employed to calculate T-cell exclusion and dysfunction scores while predicting patient responsiveness to immune checkpoint inhibitor therapies.^[24,25]^

### Mice and treatment

Trp53^fl/fl^ and Alb-Cre mice with a C57BL/6 background were purchased from Jackson Laboratory. Trp53^fl/fl^ mice were crossed with Alb-Cre mice to generate liver conditional Trp53 knockout (Trp53 cKO) mice. Sleeping beauty transposase (SB100) and transposon pT3-Neo-EF1a-GFP plasmids were purchased from Addgene. cDNA of the mouse Myc gene was cloned into the transposon vector through the MluI and SpeI restriction enzyme sites, thus producing the pT3-Neo-EF1a-Myc plasmid. Next, Kras^G12D^ fragments were obtained by PCR cloning of mouse cDNA. Subsequently, the Myc and Kras^G12D^ transposon plasmid (pT3-Myc-Kras^G12D^, pTMK) was generated *via* the AscI and NotI restriction sites.

The method for constructing an orthotopic liver tumor model in mice has been previously described.^[18]^ Briefly, plasmids for hydrodynamic tail vein (HDTV) injection were prepared with an EndoFree-Maxi Kit (Qiagen). For HDTV injection, a 30 μg DNA mixture (5 : 1 ratio of transposon to transposase-encoding plasmid) was suspended in 0.9% saline solution at a final volume equal to 10% of the body weight of the mice and was then injected into 8-week-old male Trp53 cKO mice *via* the tail vein within 5-7 s.

Ten days after injection, mice injected with pTMK were randomly assigned to different groups. To evaluate the efficacy of drug treatments in inhibiting tumor growth *in situ*, the mice were treated daily with FAK inhibitor (IN10018, 25 mg/ kg, oral gavage) until their demise. The effectiveness of this treatment was measured by analyzing survival times and tumor weights.

### Cell derived xenograft (CDX)

HCC cell lines Hepa1-6 (C57BL/6J syngeneic) and H22 (BALB/c syngeneic) were expanded in complete DMEM (Corning) supplemented with 10% fetal bovine serum (FBS, Gibco) and 1% penicillin-streptomycin (Corning) under standard culture conditions (37°C, 5% CO_2_). At 80-90% confluence, adherent cells were enzymatically dissociated using 0.25% trypsin-EDTA (Gibco), neutralized with serum-containing medium, and centrifuged at 500 ×g for 3 min. Pelleted cells were washed twice in sterile phosphate-buffered saline (PBS, pH 7.4) and resuspended in a chilled 1:1 mixture of DMEM and growth factor-reduced Matrigel matrix (Corning).

For tumor implantation, 1×10^6^ cells in a 100 μL suspension were injected subcutaneously into the right flank of 6-week-old male C57BL/6J (for Hepa1-6) or BALB/c (for H22) mice. Tumor growth was monitored daily until volumes reached 100 mm^3^ twelve days after injection, calculated as tumor volume = ½ length × width^2^. At this endpoint, mice were randomized into treatment cohorts. The FAK inhibitor (IN10018) was administered daily *via* oral gavage at 12.5 mg/ kg in 100 μL 0.5% nastrol 250 HX vehicle. Anti-PD-1 monoclonal antibody (clone RMP1-14, Bio X Cell) was delivered intraperitoneally at 10 mg/ kg in 100 μL PBS twice weekly. Anti-VEGFR2 mAb (clone DC101, Bio X Cell) was administered intraperitoneally at 10 mg/kg in 100 μL PBS twice weekly. Tumor dimensions were measured every 3 days using digital calipers. Animals were euthanized *via* cervical dislocation upon meeting endpoint. Excised tumors were weighed and processed for histopathological or molecular analyses.

### Reverse transcription and quantitative real-time PCR (RT-qPCR)

Total RNA of tissue samples was isolated using the Trizol RNA Isolation kit (Invitrogen). The First-Strand cDNA Synthesis SuperMix (TransGen Biotech) was used for reverse transcription from total RNA. Expression of different genes was tested with corresponding primers on qTOWER^3^(Analytik Jena) by TransStart Top Green qPCR SuperMix (TransGen Biotech). Relative expression levels were calculated by the comparative Ct approach with 18S rRNA as an internal control. Following primers sequences used for the queried genes are provided. *18S rRNA-Fw*: 5’-GTAACCCGTTGAACCCCATT-3’. *18S rRNA-Rv*: 5’-CCATCCAATCGGTAGTAGCG-3’. *Acta2-Fw*: 5’-ATCACCATCGGAAATGAACG-3’. *Acta2-Rv*: 5’-CTGGAAGGTGGACAGAGAGG-3’. *Col1a1-Fw*: 5’-GCAAGAGGCGAGAGAGGTTT-3’. *Col1a1-Rv*: 5’-GACCACGGGCACCATCTTTA-3’. *Timp1-Fw*: 5’-CAGATACCATGATGGCCCCC-3’. *Timp1-Rv*: 5’-TATGACCAGGTCCGAGTTGC-3’. *Tgfb1-Fw*: 5’-GTAACCCGTTGAACCCCATT-3’. *Tgfb1-Rv*: 5’-CCATCCAATCGGTAGTAGCG-3’. *Pecam1-Fw*: 5’-TTCAGCGAGATCCTGAGGGT-3’. *Pecam1-Rv*: 5’-CGCTTGGGTGTCATTCACGA-3’. *Icam1-Fw*: 5’-AGATCACATTCACGGTGCTGG-3’. *Icam1-Rv*: 5’-GCTTTGGGATGGTAGCTGGA-3’. *Vcam1-Fw*: 5’-CCCTTGCTGAATGCAAGGA-3’. *Vcam1-Rv*: 5’-TGGGACCATTCCAGTCACTTC-3’. *Vegfa-Fw*: 5’-CCACGTCAGAGAGCAACATCA-3’. *Vegfa-Rv*: 5’-TCATTCTCTCTATGTGCTGGCTTT-3’.

### Western blot

The protein samples were extracted from cell lines or animal tissues with Radio-Immunoprecipitation Assay Buffer (RIPA) lysis buffer (Solarbio). The quantitation of protein amounts was performed with the Bicinchoninic Acid (BCA) kit (Thermo Fisher Scientific). Then the samples were mixed with 4×loading buffer for electrophoresis. After transferring, the samples were incubated with primary antibodies at 4 °C overnight. The Goat anti-rabbit IgG secondary antibody (CST, Cat#7074) and the Goat anti-mouse IgG secondary antibody (CST, Cat#7076) were used for 1 h incubation at room temperature. The blotting membranes were excited by enhanced chemiluminescence (ECL) reagent (Lablead) and the exposure was procedured with Amersham ImageQuant™ 800 (Cytiva). The primary antibodies anti-FAK (CST, Cat#3285), anti-p-FAK (Tyr397; Thermo Fisher Scientific, Cat#700255), anti-α-SMA (Selleck, Cat#F2514), anti-COL1A1 (Selleck, Cat#F1228) and anti-Actin (Santa Cruz, Cat#sc-47778) were used.

### Flow cytometry

Mouse tumors were washed with ice-cold PBS and cut into small pieces, which were then subjected to enzymatic digestion with collagenase D and DNase I (Roche). Cells were filtered through a 100-μm filter and washed with PBS. To extract immune cells, the cell pellet was resuspended in a 40% Percoll gradient and then centrifuged 20 min at 800 × g with a “no brake” deceleration step. The cell pellet at the bottom was subjected to flow cytometry. Live/ Dead staining was performed using Fixable Viability dye ZOMBIE-UV. After being washed twice with PBS, cells were incubated with mouse FcR blocking reagents (BD), then incubated with fluorescently labeled antibodies on ice for 30 min. Samples were immediately analyzed with a LSRFortessa flow cytometer (BD). Doublets were excluded with height *vs*. area dot plots, and viable cells were additionally gated through Zombie UV exclusion. Ten thousand CD45^+^ cells were further gated and collected to analyze the percentages of subpopulations. Data analysis was performed in FlowJo software.

### Immunohistochemistry (IHC)

Formalin-fixed and paraffin-embedded tissues sectioned at 4 μm were used for histological evaluation of liver tumors in a mouse model. Hematoxylin and eosin (H & E) staining, Sirius red staining and Masson staining were performed for each sample. For IHC, tissue slides were deparaffinized with xylene and rehydrated through a graded series of ethanol solutions (100%, 95%, and 70%). Subsequently, the slides were subjected to antigen retrieval by microwaving in a citric acid solution for 15 min. The primary antibodies anti-Ki67 (Abcam, Cat#ab15580), anti-FAK (CST, Cat#3285), anti-p-FAK (Tyr397; Thermo Fisher Scientific, Cat#700255), anti-CD8 (Abcam, Cat#ab217344), anti-PD-1 (Abcam, Cat#ab214421), anti-α-SMA (Servicebio, Cat#BM0002), and anti-CD31 (Servicebio, Cat#GB113151) were used. Subsequently, the slides were incubated with secondary antibodies (1:1, 100 μL for each slide; HRP-anti-rabbit IgG, ZSGB, Cat#PV-6001, or HRP-anti-mouse IgG, ZSGB, Cat#PV-6002) for 10 min at room temperature. Multispectral images were scanned with a ZEISS AXIOSCAN 7 instrument. Cells of interest were quantified in Halo v3.4 (Indica Labs) or QuPath v0.2.0. Each section was evaluated by 2 or 3 experienced pathologists.

### Statistical analysis

All statistical analyses were performed using R (https://www.r-project.org/) and GraphPad Prism 9 software. Univariable Cox proportional hazards regression analyses were performed to evaluate the association between individual prognostic factors and survival outcomes. Variables demonstrating statistical significance (*P* < 0.05) in the univariable analysis were subsequently incorporated into a multivariable Cox proportional hazards regression model to identify independent prognostic factors. Two-tailed Student’s t-tests were employed for comparisons involving two groups and one-way analysis of variance (ANOVA) was applied for analyses involving more than two groups to assess statistical significance. Survival data for mice were analyzed using the Kaplan-Meier method, with intergroup differences evaluated by log-rank tests. Statistical significance was defined as *P* < 0.05.

## Results

### High expression of FAK in HCC is associated with pathological features and poor prognosis

**Figure 1:**
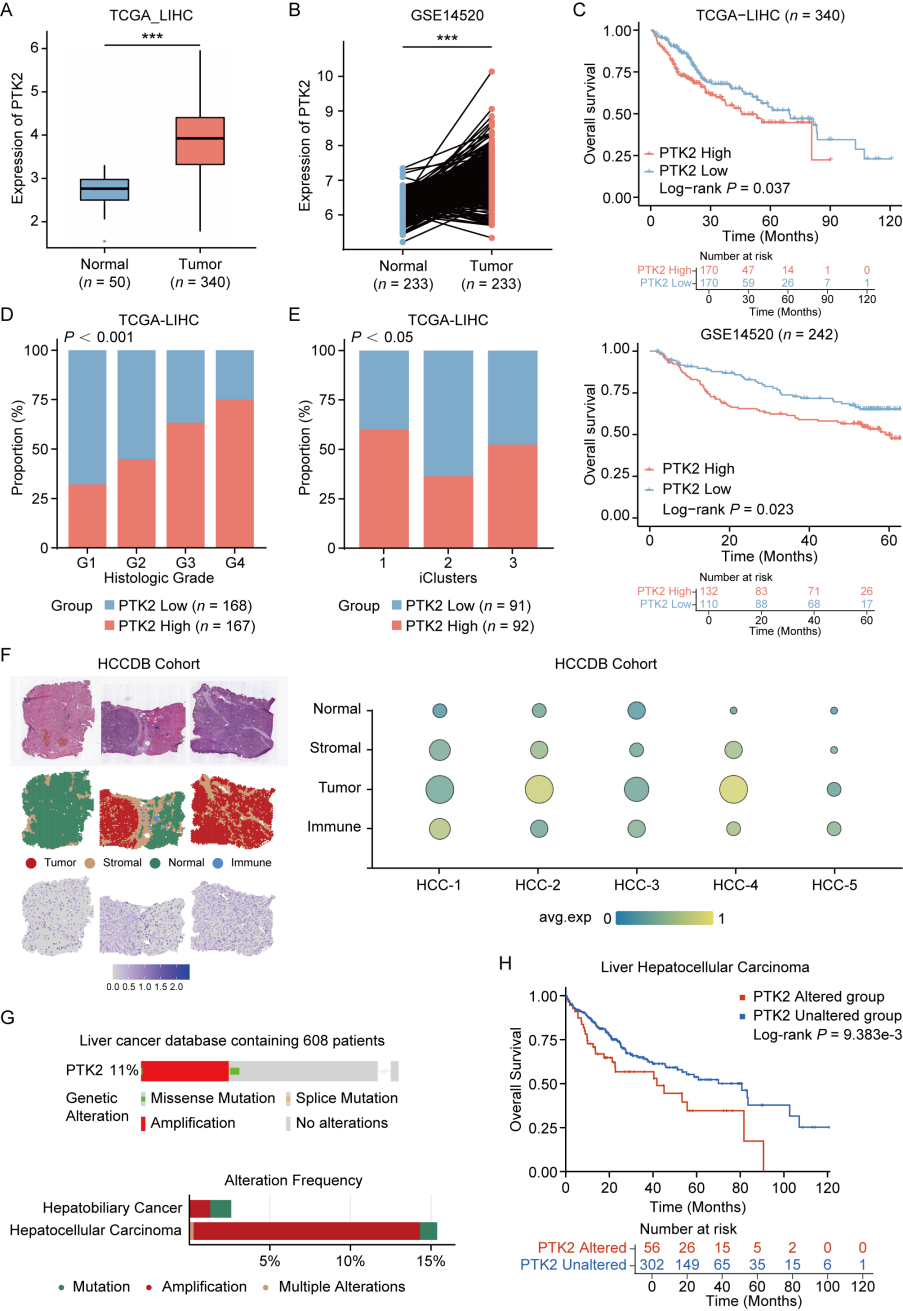
Clinical and molecular characteristics of *PTK2* expression in HCC. (A) Differential *PTK2* mRNA expression between normal liver tissue and HCC in TCGA-LIHC cohort (Normal: *n* = 50, Tumor: *n* = 340). (B) *PTK2* expression in tumor tissues versus paired adjacent non-tumorous tissues from GSE14520 cohort (*n* = 233 paired samples). (C) Kaplan-Meier survival curves stratified by *PTK2* expression value (High *vs*. Low) in TCGA (upper panel) and GSE14520 (lower panel) cohorts. (D) Association between *PTK2* expression levels (High/Low) and histological tumor grades (G1-G4) in HCC (P < 0.001 by Chi-square test). (E) Distribution of *PTK2* expression levels across molecular HCC subtypes (*P* < 0.05 by Chi-square test). (F) Spatial transcriptomic analysis of *PTK2* expression in HCC specimens from HCDDB cohort. (G) *PTK2* genomic alteration frequency in HCC and HBC. (H) Prognostic implications of *PTK2* genomic alterations across multi-omics HCC cohorts (cBioPortal), analyzed by Kaplan-Meier survival modeling. Results in each group were presented as mean ± SEM. ***P < 0.001. HCC: hepatocellular carcinoma; TCGA-LIHC: The Cancer Genome Atlas-Liver Hepatocellular Carcinoma; SEM: standard error of mean; HCDDB: Integrative molecular database of hepatocellular carcinoma; HBC: hepatobiliary cancer.

To investigate the pathogenesis of FAK in PLC, we obtained a large cohort of PLC patients from TCGA database, containing 340 tumors and 50 corresponding normal tissues. Analysis demonstrated that of *PTK2* messenger RNA (mRNA) levels were significantly elevated in tumor specimens, compared to normal samples (Figure 1A). Furthermore, a similar expression pattern was also observed in paired HCC/normal tissues from the GSE14520 dataset (*n* = 233, Figure 1B). Next, we analyzed the correlation of *PTK2* expression with pathological features of patients with PLC. The results showed that high *PTK2* expression positively correlated with short overall survival time of patients (OS, Figure 1C) and advanced histopathological grades, with G3/G4 tumors predominating in *PTK2*-positve patients (Figure 1D). *PTK2* serves as an independent prognostic factor for patients with liver cancer (Table 1). In a previous study on TCGA-LIHC data, patients were molecularly categorized into three subtypes based on their expression profiles.^[26]^ In summary, iCluster1 was characterized by higher tumor grade and macrovascular invasion, while iCluster2 was associated with low-grade tumors and less microvascular invasion. iCluster3 was characterized by high chromosomal instability, with iCluster1 patients having the worst prognosis and iCluster2 patients having the best prognosis. Interestingly, we found that patients with higher *PTK2* expression also had a higher proportion of the iCluster1 molecular subtype (Figure 1E), which further indicated that high *PTK2* expression is associated with poor clinical features in HCC patients. Finally, spatial transcriptomic profiling through HCCDB V.2^[27]^ confirmed tumor-specific *PTK2* upregulation, with predominant expression localized to malignant hepatocytes and stromal compartments (Figure 1F). To support our findings, we also analyzed the gene alteration of *PTK2* in patients with HCC *via* cBioPortal website (https://www.cbioportal.org/).^[28, 29, 30]^ The results showed that *PTK2* gene was predominantly amplified, accounting for 8% of HCC patients and this gene alteration predicted worse prognosis of patients (Figure 1G, 1H). Collectively, these findings suggested that *PTK2* was identified as a biomarker of tumor progression linked to poor differentiation and adverse clinical outcomes in HCC.

### FAK suppresses CD8^+^ T Cell infiltration and predicts resistance to anti-PD-1 therapy in HCC

**Figure 2:**
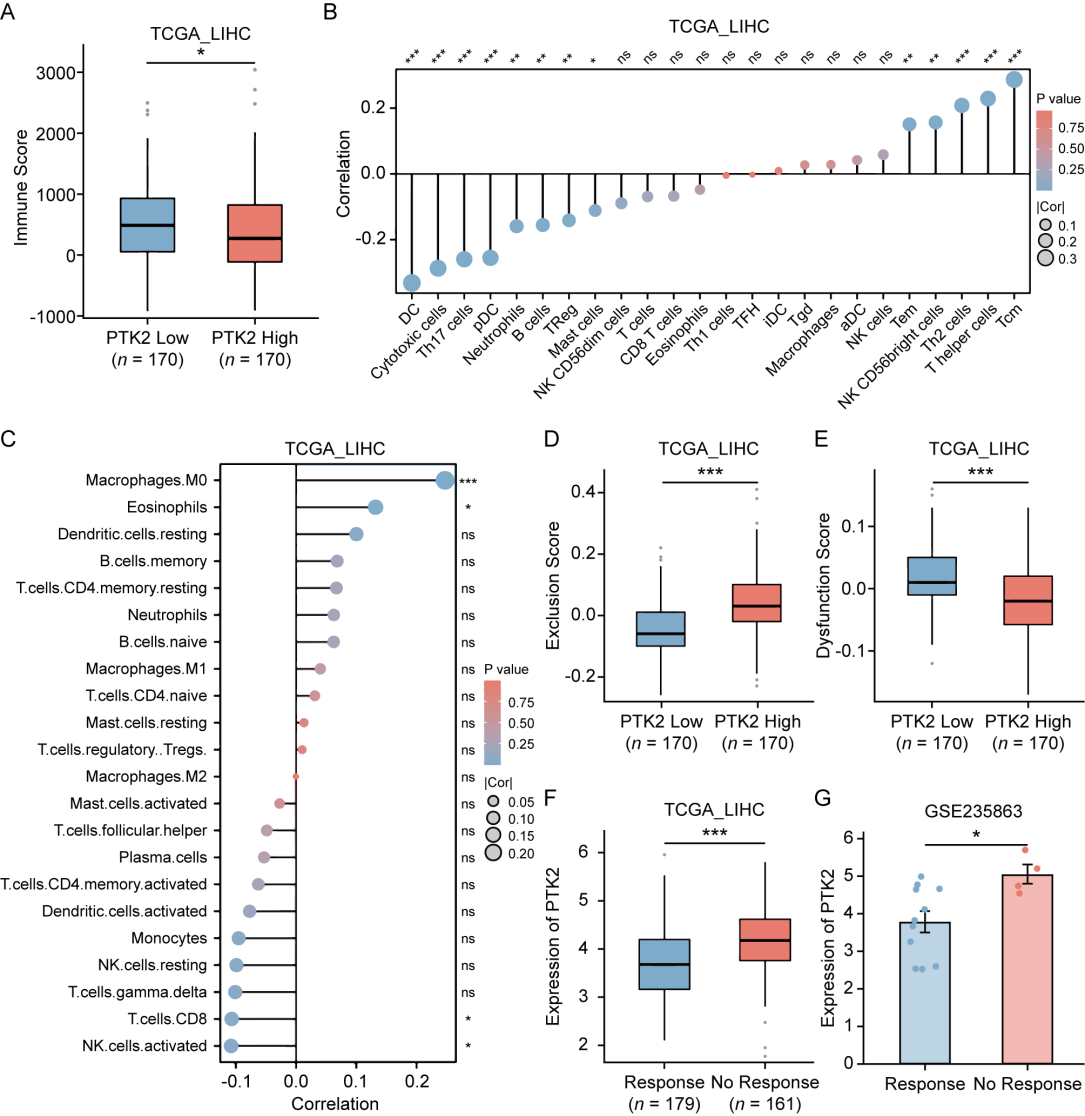
Association of *PTK2* expression with tumor immune microenvironment and ICI response in HCC. (A) Immune scores calculated by the ESTIMATE algorithm in TCGA-LIHC cohort, stratified by *PTK2* expression levels (*n* = 170). (B, C) Spearman’s correlation analysis between *PTK2* mRNA expression and immune cell infiltration levels quantified by ssGSEA (B) or CIBERSORT (C) in TCGA-LIHC cohort. (D, E) Comparison of tumor immune exclusion score (D) and T cell dysfunction score (E) between *PTK2*-high and *PTK2*-low HCC subgroups. (F) Differential *PTK2* mRNA expression between TIDE-predicted ICIs responders and non-responders in TCGA-LIHC cohort (Responders: *n* = 179 *vs*. Non-responders: *n* = 161). (G) Differential *PTK2* expression between responders and non-responders to anti-PD-1 plus lenvatinib combination therapy in GSE235863 cohort (Responders: *n* = 11 vs. Non-responders: *n* = 4). Results in each group were presented as mean ± SEM. *P < 0.05, **P < 0.01, ***P < 0.001. SEM: standard error of mean; ICI: immune checkpoint inhibitor; HCC: hepatocellular carcinoma; TCGA-LIHC: The Cancer Genome Atlas-Liver Hepatocellular Carcinoma; ssGSEA: single-sample gene set enrichment analysis; TIDE: Tumor Immune Dysfunction and Exclusion.

We further investigated the association between FAK (*PTK2*) expression and the immune microenvironment in HCC. Analysis of the TCGA-LIHC cohort using the ESTIMATE algorithm revealed significantly lower immune scores in *PTK2*-positve patients (Figure 2A). Subsequent immune deconvolution *via* ssGSEA and CIBERSORT demonstrated *PTK2* expression was negatively correlated with cytotoxic immune cells and positively correlated with suppressive myeloid cells (Figure 2B, 2C). Given the critical role of CD8^+^ T cell infiltration in ICI efficacy,^[31,32]^ we evaluated *PTK2*’s impact on therapeutic response using the TIDE algorithm. *PTK2*-high tumors exhibited elevated Exclusion Scores (Figure 2D), reflecting reduced immune cell infiltration, while *PTK2*-low tumors showed higher Dysfunction Scores (Figure 2E), indicative of T cell exhaustion potentially reversible by PD-1 blockade.^[33]^ Clinically, TIDE-predicted non-responders to anti-PD-1/ PD-L1 therapy had higher *PTK2* expression (Figure 2F), a consistent phenomenon confirmed in the GSE235863 cohort where non-responders to anti-PD-1 combined Lenvatinib therapy also displayed elevated *PTK2* levels (Figure 2G). These findings collectively demonstrated *PTK2* overexpression may drive immune suppression by inhibiting cytotoxic lymphocyte infiltration, suggesting inhibition of *PTK2* provides HCC patients more likelihood to benefit from anti-PD-1 therapy.

### FAK inhibitor IN10018 suppresses tumor progression in orthotopic PLC models

**Figure 3:**
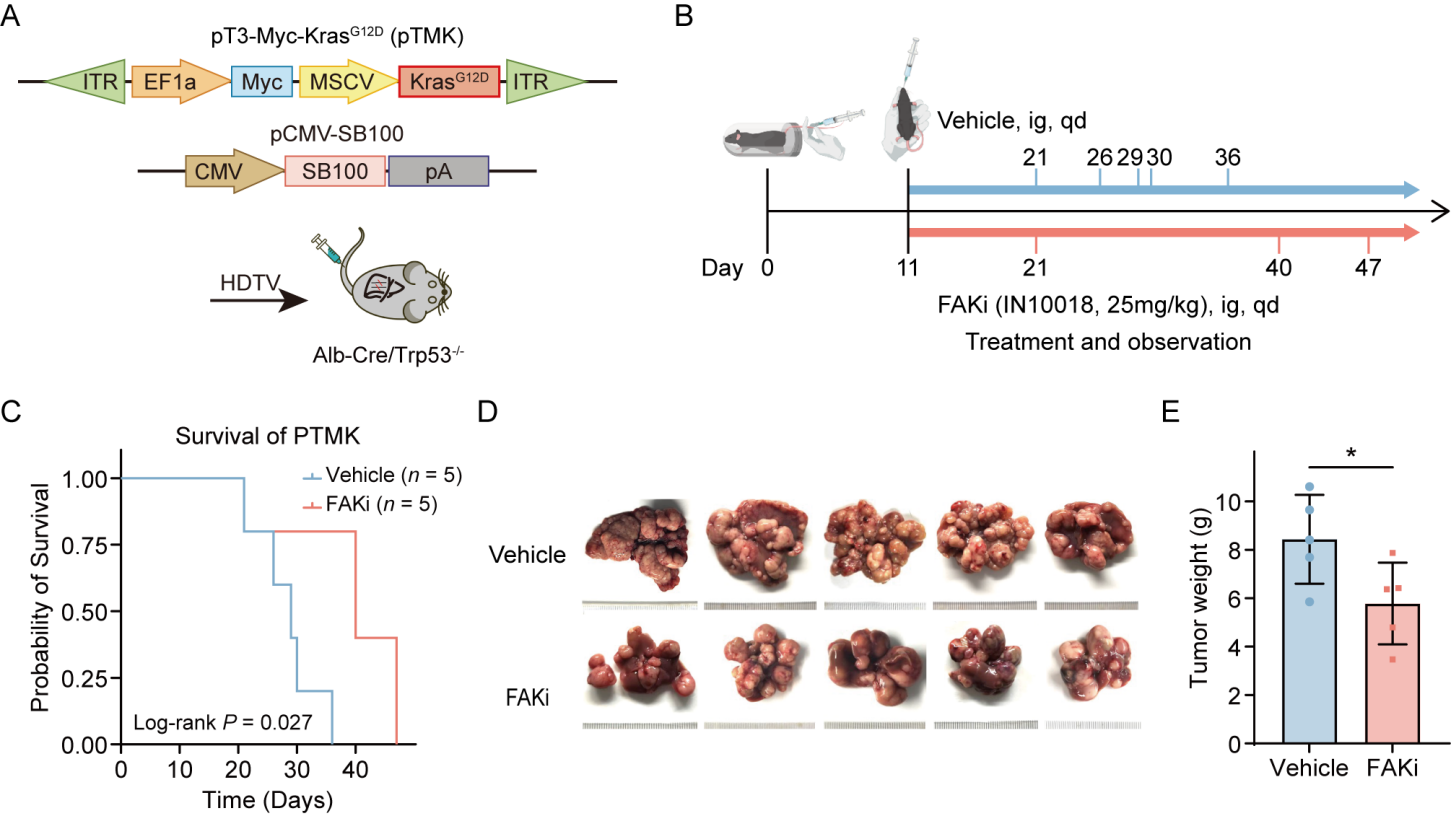
Therapeutic efficacy of FAK inhibition in a genetically engineered mouse model of PLC. (A) Schematic illustrating the establishment of the transgenic PLC model: transposable vectors pTMK (encoding Myc and KrasG12D) combined with the SB100 transposase vector were delivered into Alb-Cre × Trp53fl/fl mice *via* HDTVI. (B) Experimental design timeline: Mice were randomized into vehicle control and FAK inhibitor treatment groups (*n* = 5/group). FAK inhibitor (25 mg/kg) or vehicle administered daily by oral gavage from Day 11 post-HDTVI until the mice died. (C) Kaplan-Meier survival curves demonstrating prolonged survival in the FAK inhibitor-treated cohort versus controls. (D) Representative photos of pTMK tumors generated in FAKi group and control groups. The ruler tick marks show mm. (E) The bar plots show the tumor weight (*n* = 5). Results in each group were presented as mean ± SEM. *P < 0.05. FAK: focal adhesion kinase; SEM: standard error of mean; PLC: primary liver cancer.

The amplification of the oncogene MYC, KRAS mutations, and deletions of the tumor suppressor gene TP53 are prevalent genetic alterations in liver cancer patients, often occurring concurrently with one another. To validate our findings from the database, we well-established the orthotopic liver cancer model to replicate these significant genomic alterations. We generated a transposon vector co-expressing mouse Myc and mouse KRAS^G12D^ (pTMK), in which Myc was driven in an E2F-dependent manner and KRAS^G12D^ was regulated by a strong MSCV promoter. Liver-specific knockout of Trp53 is achieved through Albumin-Cre mediated recombination. We employed hydrodynamic tail vein (HDTV) injection to co-deliver the transposon vector pT3-Myc-Kras^G12D^ with the sleeping beauty transposase plasmid (SB100) into Trp53 liver conditional mice, leading to integration of transposable elements into hepatocyte genomic DNA of mice, referred to as the pTMK model (Figure 3A).^[18]^ According to earlier descriptions, this combinatorial technique caused extensive liver damage and accelerated liver cancer. Around the twentieth day after injection, the phenomenon of liver cancer leading to mortality started to emerge. The pTMK cohort demonstrated a median survival of 30-40 days, with all mice ultimately developing *in situ* liver tumors, in sharp contrast to control mice receiving the pT3 empty vector, which had a survival exceeding 180 days. Histopathological analysis confirmed features of HCC including positive hepatocyte staining and architectural patterns that reflected the pathological heterogeneity of PLC in humans.

Daily oral administration of the FAK inhibitor IN10018 significantly prolonged survival time, compared to vehicle-treated controls (Figure 3B, 3C). Therapeutic efficacy was further confirmed by marked suppression of tumor growth in IN10018-treated mice, as evidenced by reduced liver weight (Figure 3D, 3E). These preclinical results substantially corroborate clinical observations, demonstrating that FAK inhibition attenuates PLC progression in genetically engineered models recapitulating key oncogenic drivers in human PLC.

### FAK inhibitor suppresses HCC proliferation, fibrosis, and angiogenesis while enhancing CD8^+^ T Cell infiltration in mouse models

**Figure 4:**
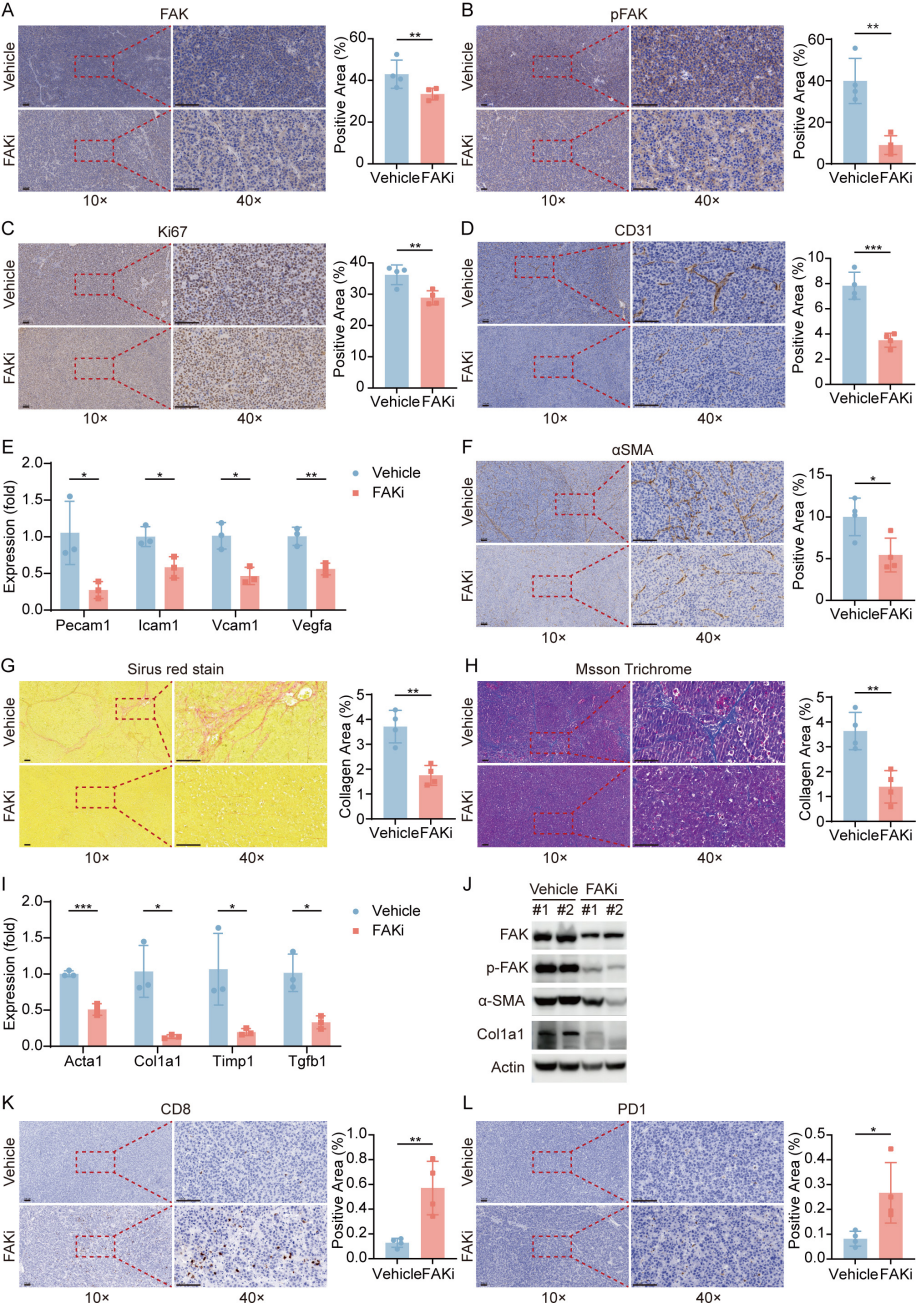
FAK Inhibition reprograms tumor microenvironment *via* suppressing proliferation, angiogenesis, and restoring anti-tumor immunity. (A-D) Representative IHC images and quantification of area performed by FAK (A), phospho-FAK (B), Ki67 (C), CD31 (D) immunohistochemistry on liver tumor tissues of mice treated with vehicle or FAK inhibitor. (E) qPCR was used to evaluate the expression of angiogenesis-associated markers (Pecam1, Icam1, Vcam1, Vegfa). (F) Representative IHC images and quantification of area performed by α-SMA immunohistochemistry on liver tumor tissues of mice treated with vehicle or FAK inhibitor. (G-H) Representative images and quantification of collagen area performed by Sirius red staining (G) and Masson staining (H) on liver tumor tissues of mice after treatment. (I) qPCR was used to evaluate the expression of fibrosis-related genes (Acta2, Col1a1, Tgfb1, Timp1). (J) Western blots of FAK, p-FAK, α-SMA, and Col1a1 expression in the orthotopic liver tumor tissues after treatment. (K-L) Representative IHC images and quantification of area performed by CD8 (K) and PD-1 (L). Scale bar: 100 μm. Results in each group were presented as mean ± SEM. *P < 0.05, **P < 0.01, ***P < 0.001. FAK: focal adhesion kinase; SEM: standard error of mean.

To elucidate the mechanisms underlying FAK inhibitor-mediated tumor suppression, we performed IHC assays on pTMK tumor tissue samples. As expected, FAK inhibitor IN10018 treatment significantly reduced FAK expression and phosphorylation (Figure 4A, 4B). Meanwhile, FAK inhibitor also reduced Ki67^+^ proliferating cell numbers (Figure 4C), showing evidence for its anti-proliferative effect. Consistent with our molecular subtyping findings (Figure 1E), the inhibitor concurrently decreased CD31^+^ (an angiogenesis marker) vascular density (Figure 4D) and α-SMA^+^ stromal activation areas (Figure 4F), demonstrating dual inhibitory effects on both angiogenesis and fibrotic remodeling. Consistently, expression of angiogenesis-associated markers (Pecam1, Icam1, Vcam1, Vegfa) were significantly reduced in the FAK inhibitor-treated group, but not in control group (Figure 4E). Sirius red-stained collagen regions and Masson-stained collagen fibres exhibited significant decrease in FAK inhibitor-treated tissues compared to vehicle control (Figure 4G, 4H). Furthermore, qPCR data showed reduced expression of fibrosis-related genes (Acta2, Col1a1, Timp 1, Tgfb 1) in the therapy group (Figure 4I). Western blot analysis validated a significant downregulation of fibrosis-related proteins (α-SMA and Collagen I) in the treated tumors (Figure 4J). These consistent and predominant data support our hypothesis of anti-fibrotic effects of FAK inhibition on the pTMK tumors. Collectively, our results demonstrated that FAK inhibitors significantly suppress fibrosis and angiogenesis in our orthotopic liver tumor models.

Notably, FAK inhibition markedly increased intratumoral CD8^+^ T cell infiltration(Figure 4K) and upregulated PD-1 expression (Figure 4L), aligning with the high Dysfunction Score observed in *PTK2*-low tumors (Figure 2D). This suggests that FAK inhibition may reprogram the immunosuppressive microenvironment to enhance sensitivity to anti-PD-1 therapy. Collectively, FAK inhibitor IN10018 exerts multifaceted antitumor effects by targeting cancer cell proliferation, stromal remodeling, and immune microenvironment reprogramming, providing experimental rationale for its combination with immunotherapies.

### FAK inhibitor synergizes with anti-PD-1 to enhance CD8^+^ T cell function and achieve superior tumor control

**Figure 5:**
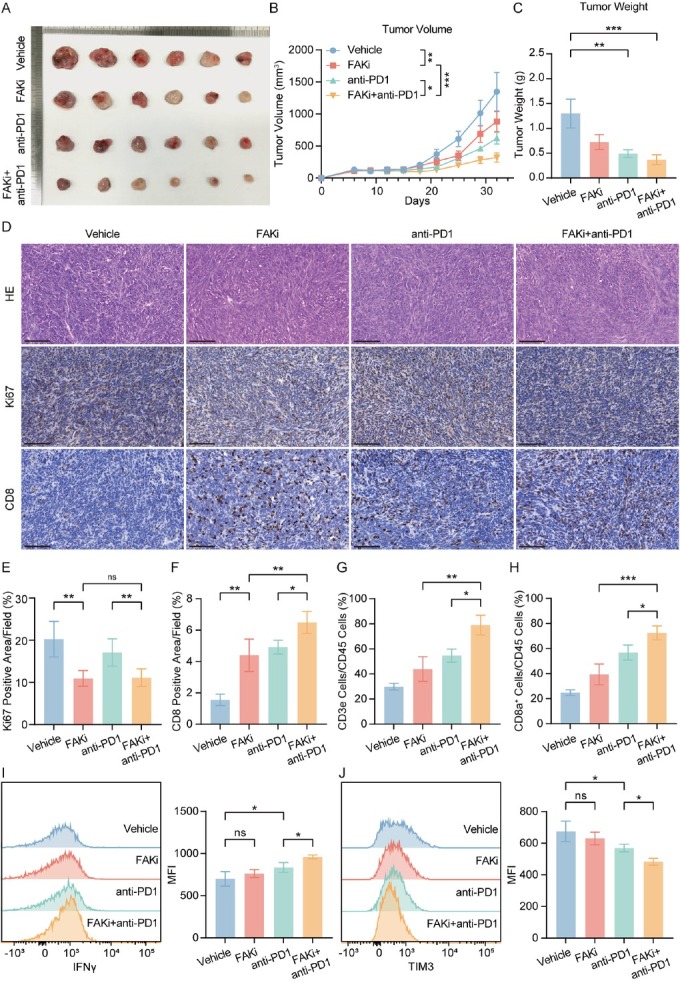
Combining FAK inhibitor and PD-1 blockade reduced tumor growth and enhanced anti-tumor effect. (A) Representative photos of Hepa1-6 subcutaneous tumors generated in FAKi group, anti-PD-1 group, combination group and control groups. The ruler tick marks show mm. (B) Volume change in mean subcutaneous implanted tumors following treatment of FAK inhibitor or anti-PD-1 beginning at the day when implanted tumor volume reached 100 mm3 (*n* = 6). (C) The bar plots show the tumor weight (*n* = 6). (D-F) Representative IHC images and quantification of area performed by Ki67 (E) and CD8 (F). Scale bar: 100 μm. (G, H) FACS analyses showed the percentage of intratumoral CD3+ (G) and CD8+ (H) of CD45+ cells (*n* = 6). (I, J) Representative flow cytometry images showed the expression of functional markers IFNγ (I) and TIM3 (J) in tumors. Results in each group were presented as mean ± SEM. *P < 0.05, **P < 0.01, ***P < 0.001. FAK: focal adhesion kinase; SEM: standard error of mean.

To validate whether FAK inhibitor-mediated CD8^+^ T cell infiltration improves the response to anti-PD-1 blockade, we established Hepa1-6 subcutaneous tumor models and treated them with FAK inhibitor (IN10018), anti-PD-1 monotherapy, or their combination from day 12 to day 32 after implantation. The combination group exhibited significantly reduced tumor volume (Figure 5A, 5B) and tumor weight (Figure 5C). Meanwhile, IHC confirmed FAK inhibitor reduced Ki67+ proliferating cell numbers and increased CD 8^+^ T cells infiltration, while combination therapy augmented this therapeutic impact (Figure 5D, 5F). Flow cytometry revealed that FAK inhibitor monotherapy increased intratumoral CD3^+^ and CD8^+^ T cell proportions, whereas combination therapy further amplified these effects (Supplementary Figure S1A, Figure 5G, 5H). Critically, combination therapy enhanced IFNγ^+^ effector function CD8^+^ T cells (Figure 5I and reduced exhausted CD8^+^ T cells marked by TIM3 (Figure 5J). In H22 subcutaneous models, while all treatment groups showed tumor suppression, combination therapy provided no additive benefit compared to monotherapies (Supplementary Figure S1B, 1C, 1D).

### FAK inhibitor/anti-PD-1 combination outperforms clinical standard anti-VEGFR-TKI regimen in preclinical HCC models

**Figure 6:**
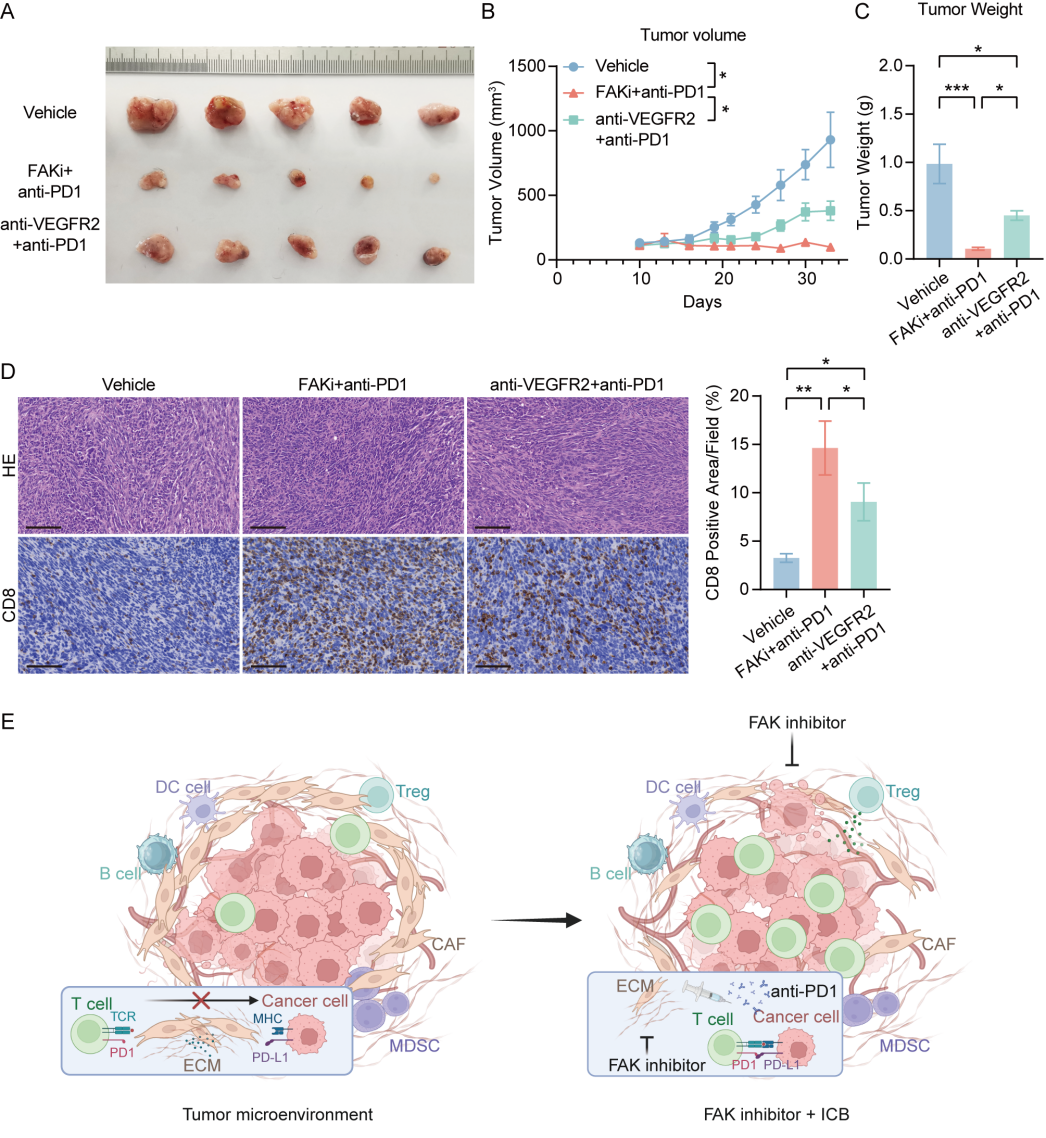
FAK inhibitor/anti-PD-1 combination outperforms clinical standard anti-VEGFR-TKI regimen in preclinical HCC models.(A) Representative photos of Hepa1-6 subcutaneous tumors generated in FAKi and anti-PD-1 combination group, anti-VEGFR2 and anti-PD-1 combination group and control groups. The ruler tick marks show mm. (B) Volume change in mean subcutaneous implanted tumors following treatment (*n* = 5). (C) The bar plots show the tumor weight (*n* = 5). (D) Representative IHC images and quantification of area performed by CD8. Scale bar: 100 μm. (E) Proposed mechanism of FAK inhibitor-mediated enhancement of anti-PD-1 efficacy. (F) The schematic figure of the combinational regimen of FAK inhibition and ICIs, created in BioRender. Results in each group were presented as mean ± SEM. *P < 0.05, **P < 0.01, ***P < 0.001. HCC: hepatocellular carcinoma; FAK: focal adhesion kinase; SEM: standard error of mean.

We further compared the therapeutic efficacy of FAK inhibitor/anti-PD-1 combination therapy with the current clinical standard anti-VEGFR-TKI/anti-PD-1 regimen. In subcutaneous tumor models, the FAK inhibitor/anti-PD-1 combination demonstrated the most pronounced tumor volume reduction (Figure 6A, 6B) and significantly decreased tumor weight compared to the anti-VEGFR-TKI/anti-PD-1 group (Figure 6C). IHC analysis further demonstrated that while both combinations enhanced intratumoral CD8^+^ T cell infiltration, the FAK inhibitor/ anti-PD-1 regimen drove more robust T cell accumulation compared to the anti-VEGFR-TKI-based therapy (Figure 6D). These findings not only confirmed FAK inhibition as a potent enhancer of anti-PD-1 efficacy but also provide critical preclinical evidence supporting clinical trials of FAK inhibitor/anti-PD-1 combinations in HCC.

## Discussion

In this study, we demonstrated that FAK inhibitor IN 10018 suppresses tumor growth in both subcutaneous and orthotopic PLC mouse models while attenuating hepatic fibrosis and angiogenesis. Mechanistically, this is at least partially mediated by promoting the infiltration of CD8^+^ T cell into the tumor tissue, suggesting the potential of FAK inhibition to mitigate resistance to immune checkpoint blockade.

In our study, both orthotopic and subcutaneous PLC models were utilized to more accurately recapitulate the pathological features observed in patients with PLC. By contrast, previous studies on liver cancer usually used subcutaneous xenografts, which inadequately mimic immune microenvironment of liver cancers. This difference was crucial to our novel findings that tumors harboring genetic alterations of MYC and KRAS were susceptible to FAK inhibition. These findings have fundamental implications for the precision therapy of patients who have such genetic alterations.

FAK acts as a central hub for oncogenic signaling, driving cancer growth and metastasis through its frequent overexpression and activation in multiple malignancies.^[15,17,34]^ Previous studies have shown that FAK can promote the development of intrahepatic cholangiocarcinoma (iCCA) by enhancing the phosphorylation of Yes-associated protein (YAP).^[35]^ Consistent with this, we found that the expression of FAK was associated with higher tumor grade and poorer prognosis in PLC patients. Furthermore, FAK plays a critical role in angiogenesis by mediating vascular endothelial growth.^[36]^ FAK also drives angiogenesis *via* VEGFR2-mediated endothelial activation.^[36, 37, 38, 39]^ Consistent with studies, our orthotopic PLC models confirmed that FAK inhibition effectively suppresses tumor vascularization. Additionally, emerging evidence highlights FAK as a key regulator of tumor microenvironment remodeling through chemokine secretion and immune evasion mechanisms.^[40]^

FAK can indirectly modulate immunosuppression by promoting Treg differentiation *via* PI3K/AKT/JAK/ STAT3 and p38/JNK signaling pathways, which facilitates macrophage M2 polarization and subsequently inhibits T cell function.^[41]^ In some cases, FAK inhibition has also

been shown a decrease in the numbers of macrophages, monocytic myeloid-derived suppressor cells (M-MDSCs) and granulocytic myeloid-derived suppressor cells (G-MDSCs) in tumors.^[17,42,43]^ Consistent with these studies, our analyses demonstrated a significant decrease in intratumoral CD11b^+^Gr1^+^ MDSCs and a reduction in CD274 expression of MDSCs after FAK inhibitor treatment. These data suggest that FAK inhibitors may enhance the activity of CD8^+^ T cells by diminishing the invasion of MDSCs or reversing the polarization of tumor-associated macrophages (TAMs), reprogramming immune microenvironment. Collectively, FAK inhibitor IN10018 demonstrated potent anti-tumor efficacy in our PLC models, mediated through integrated multiple mechanisms driving CD 8^+^ T cell infiltration—particularly the interplay between stromal remodeling, angiogenesis inhibition and myeloid cell-mediated TIME reprogramming.

Although anti-PD-1/PD-L1 monotherapy achieved some tumor response in the early stages of clinical trials, it did not demonstrate superior efficacy compared to multi-target therapy.^[10,44,45]^ Identifying reliable predictive biomarkers and developing novel combination strategies remain critical challenges for optimizing ICI efficacy.^[14]^ However, most HCC exhibit immunosuppressive microenvironments that restrict CD8^+^ T cell infiltration,^[46, 47, 48]^ rendering them immunologically “cold” tumors devoid of PD-1/PD-L1 axis activity. In our study, FAK inhibitor treatment upregulated PD-1 expression in orthotopic PLC models, consistent with our TIDE analysis revealing higher T cell dysfunction cytotoxic scores score in *PTK2*-low tumors. While these infiltrating T cells display early exhaustion phenotypes, concurrent anti-PD-1 therapy may reverse their dysfunctional state, as evidenced by enhanced effector function and tumor control in our models. The finding further supported by enhanced antitumor immunity when combining FAK inhibitors with T-cell co-stimulators.^[49,50]^ This mechanistic synergy provides a rationale for timing FAK inhibition with PD-1 blockade to exploit the window of maximal T cell receptivity to checkpoint reactivation.^[51,52]^ Such insights will be critical for optimizing combination strategies and identifying patient subgroups most likely to benefit from FAK/anti-PD-1 therapy.

Although ongoing clinical trials primarily evaluate defactinib (a dual FAK/PYK2 inhibitor) in combination with anti-PD-1 agents, there is no clinical evidence supporting superiority of dual FAK/PYK2 inhibition over FAK-specific targeting.^[17]^ Our results suggest that the FAK inhibitor/anti-PD-1 regimen achieved comparable or superior therapeutic outcomes relative to current atezolizumab (anti-PD-L1) and bevacizumab (anti-VEGF) combination therapy in murine models, providing preliminary proof of the potential for clinical translation.

## Conclusion

This study establishes FAK overexpression is correlated with advanced tumor grade and unfavorable clinical prognosis in HCC. Preclinically, the FAK inhibitor IN10018 demonstrated consistent antitumor efficacy across orthotopic and subcutaneous HCC models, synergizing with anti-PD-1 therapy to enhance CD8^+^ T cell-dependent immune control, leading to tumor suppression. These provides a compelling rationale for clinical evaluation of FAK/anti-PD-1 combinations in HCC patients unresponsive to existing anti-angiogenic/immunotherapy regimens.

## Supplementary Information

Supplementary materials are only available at the official site of the journal (www.intern-med.com).

## Supplementary Material

Supplementary Material Details
