# Supplementary material for: Focal adhesion kinase inhibition enhances response to checkpoint immunotherapy in hepatocellular carcinoma
Source: J Transl Int Med. 2025 Dec 12;14(1):64–78. doi: 10.1515/jtim-2025-0051 (PMC12916278; doi:10.1515/jtim-2025-0051)
Supplement: Supplementary file 1 — Supplementary Material Details [file jtim-2025-0051_sm.pdf]

Supplementary Materials

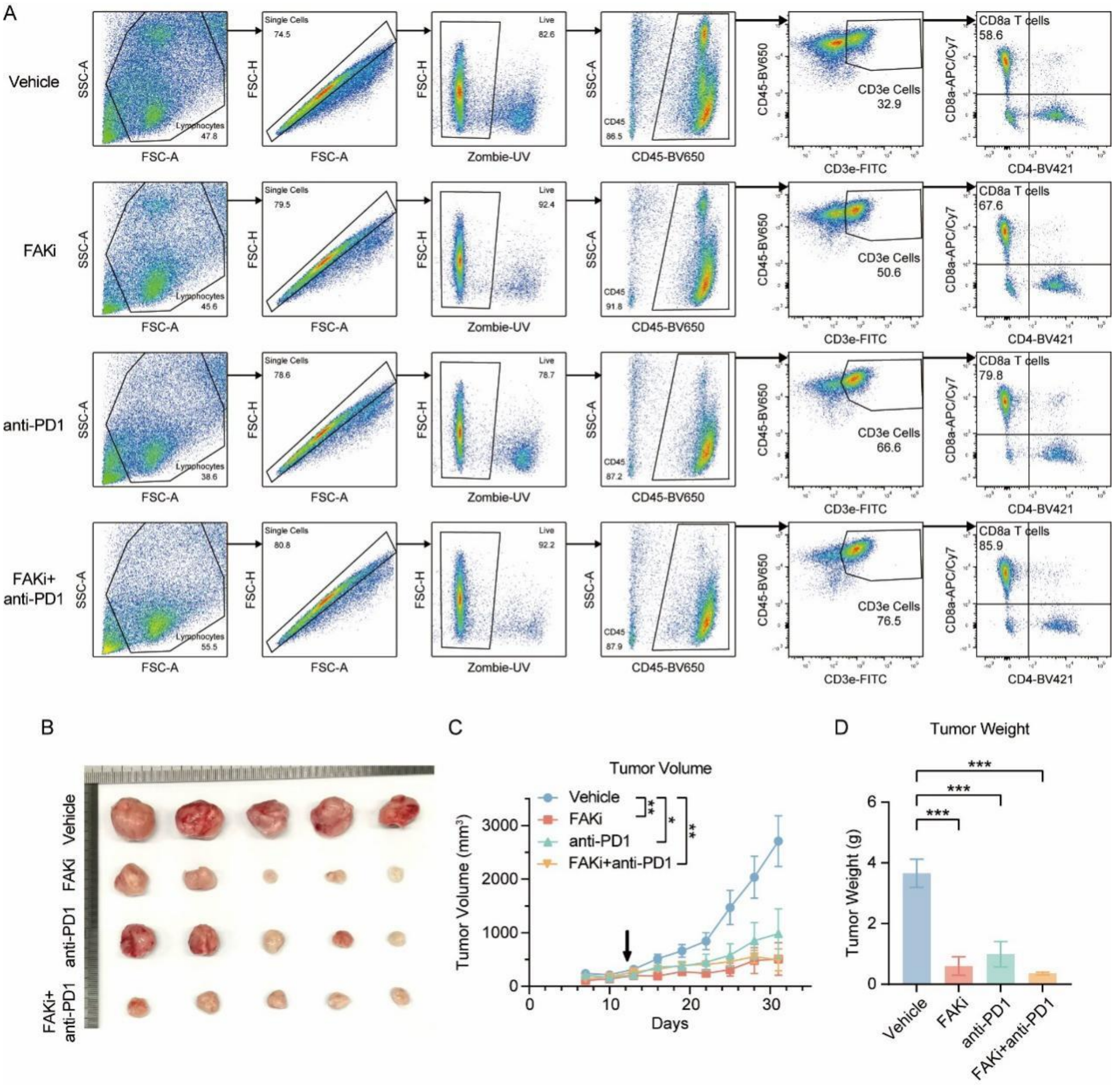

Supplementary Figure S1: Combining FAK inhibitors and PD-1 blockade reduced tumor growth.

(A) Representative plots from all presented populations. (B) Representative photos of H22 subcutaneous tumors generated in FAKi group, anti-PD1 group, combination group and control groups. The ruler tick marks show mm. (C) Volume change in mean subcutaneous implanted tumors following treatment with FAK inhibitor or anti-PD1 (*n* = 5). (D) The bar plots show the tumor weights (*n* = 5). (Results in each group were presented as mean ± SEM. $^{*}P < 0.05$, $^{**}P < 0.01$, $^{***}P < 0.001$).
